# Long‐Term Effectiveness of a Stent‐Less Strategy With Drug Coated Balloon in Coronary Artery Disease: 3‐Year Follow‐Up of a Prospective All‐Comers Observational Study

**DOI:** 10.1002/clc.70189

**Published:** 2025-07-31

**Authors:** Ludovic Meunier, Simon Eccleshall, Ronan Bakdi, Matthieu Godin, Géraud Souteyrand, Benoît Mottin, Yann Valy, Christian Benoit, Vincent Lordet, Virginie Laurençon, Antoine Milhem, Matthias Waliszewski, Caroline Allix‐Béguec

**Affiliations:** ^1^ Cardiology Department Centre Hospitalier la Rochelle la Rochelle France; ^2^ Norfolk and Norwich University Hospital Norwich UK; ^3^ Cardiology Department Clinique St‐Hilaire Rouen France; ^4^ Département de Cardiologie, CHU Clermont‐Ferrand, ISIT, CaVITI, CNRS (UMR‐6284) Université d'Auvergne Clermont‐Ferrand France; ^5^ Clinical Trials Unit Centre Hospitalier la Rochelle la Rochelle France; ^6^ Medical Scientific Affairs B. Braun Melsungen AG Berlin Germany

**Keywords:** angioplasty, coronary artery disease, DCB‐based strategy, drug‐coated balloon, drug‐eluting stent, patient outcome assessment, percutaneous coronary intervention

## Abstract

**Introduction:**

Drug‐eluting stent (DES) angioplasty is the gold standard treatment for coronary lesions. Drug‐coated balloon (DCB) is an option for in‐stent restenosis, and has also shown promise for small‐calibre coronary artery disease. We evaluated the 3‐year effectiveness of a decision algorithm for percutaneous coronary intervention (PCI) that favoured a stent‐less strategy (SLS) in primary angioplasty.

**Methods:**

All patients who underwent angioplasty during 1 year were included in a prospective observational study. Patients eligible for SLS first underwent scoring balloon followed by DCB angioplasty or DES in case of mandatory bailout. Patients not eligible for SLS were unstable patients who underwent conventional drug‐eluting stenting. The metal index, stent burden, was calculated by stent length divided by the total lesion length. A 36‐month follow‐up recorded target lesion revascularization (TLR).

**Results:**

Patients eligible for SLS represented 85% (*n* = 840) of patients who underwent PCI. TLR was required in 2.6% and 6% of patients in the DCB‐only and bailout‐DES groups, respectively. Median metal index was 0.25 (IQR: 0.5) in patients with TLR. There was a difference between TLR–free survival distributions in the DCB‐only and bailout‐DES groups (*p* = 0.016).

**Conclusions:**

The SLS based on a combination of scoring balloon and DCB was effective at 3 years with a low rate of TLR. This rate was higher in patients with stent burden.

**Trial Registration:** This study was registered with clinicaltrials. gov (NCT03893396, first posted on March 28, 2019).

## Graphical abstract

The effectiveness of percutaneous coronary interventions following a decision tree proposing a stent‐less strategy in stable patients with coronary artery disease is illustrated by Kaplan‐Meier curves of target lesion revascularisation, the study endpoint. DCB: Drug Coated Balloon; DES: Drug Eluting Stent; SCBA: scoring balloon angioplasty.

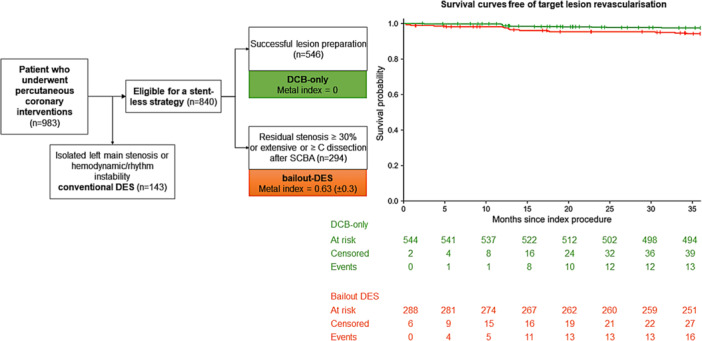

**Abbreviations**
DCB: drug‐coated balloon
DES: drug‐eluting stent
LVEF: left ventricular ejection fraction
MACE: major adverse cardiac events
NSTEMI: non‐ST myocardial infarction
PCI: percutaneous coronary intervention
SLS: stent‐less strategy
STEMI ST: myocardial infarction
TLR: Target lesion revascularization

## 1 Introduction

The revascularization of coronary lesions without stenting has developed in recent years by using Drug Coated Balloons (DCB). After the initial indication to treat in‐stent restenosis [1], DCB angioplasty is gradually being used to treat de novo lesions such as small vessels [2], bifurcations [3], highly calcified lesions [4] and chronic total occlusions [5].

The stent‐less strategy is based on lesion preparation to maximize the effect of the DCB angioplasty used afterwards [6]. It aims at simplifying the complexity and at leaving nothing behind. Some coronary lesions are called complex lesions. It is not the lesion but the technique of revascularisation using stents that is complex. For example, in the treatment of bifurcations, sequential revascularization without stenting will simplify the procedure by avoiding carina shift, proximal optimisation technique, rewiring of the side branch through distal struts, kissing and DK crushing for example. The leave nothing behind concept is an attempt to reduce subsequent thrombotic events by leaving no metallic material in the arteries. To prevent early thrombosis, a dual antiplatelet regimen is combined with stenting, but this strategy may increase risk in patients at high risk of bleeding. A dual antiplatelet regimen is prescribed after PCI and lasts several months. Retrospective studies suggested that single antiplatelet therapy in high bleeding risk patients revascularized by DCB could reduce the rate of haemorrhagic events [7], without increasing the number of ischaemic events [8]. A randomized controlled trial in elective coronary revascularisation is currently underway to validate this approach (DEBATE). The leave nothing behind concept is also to avoid the risk of very late stent‐related events in high‐risk ischemic patients revascularized by stenting. In the absence of a stent, this continuous ischemic risk theoretically disappears. Studies comparing long‐term outcome of DCB versus DES showed a continued risk of major adverse cardiac events (MACE) in the drug‐eluting stent groups, whereas no additional MACE was reported in the DCB groups after 1 year [9, 10].

In our centre, we have implemented the stent‐less strategy using balloon scoring for the lesion preparation followed by paclitaxel‐coated balloon angioplasty in all eligible patients. We collected data on TLR at 3 years to ensure the effectiveness of this stent‐free strategy over time.

## 2 Materials & Methods

### 2.1 Study Design

Prospective all‐comers observational study to evaluate 3‐year effectiveness of a stent‐less strategy for percutaneous coronary intervention (PCI).

### 2.2 Setting

PCI were performed in an interventional cardiology unit of a public hospital staffed with five interventional cardiologists with different levels of experience (three seniors, one junior, and one part‐time cardiologist) in coronary angioplasty and stenting.

### 2.3 Participants

The inclusion criteria were as follows: All consecutive patients in whom coronary angioplasty was performed, i.e. acute coronary syndrome non‐ST myocardial infarction (NSTEMI) or ST myocardial infarction (STEMI), chronic coronary syndrome including angina, silent ischemia, ischemic heart disease, were eligible. The exclusion criteria were patients < 18 years of age, unstable patient and/or patient treated for left coronary main trunk stenosis with conventional DES angioplasty, pregnancy, legally protected patients or deprived of liberty, and refusal to participate.

### 2.4 Percutaneous Coronary Intervention

If the patient was hemodynamically or rhythmically unstable and/or was being treated for left coronary main trunk stenosis, conventional DES angioplasty was performed. Unstable patients were those in shock, with ventricular hyperexcitability (ventricular tachycardia, ventricular fibrillation), or at risk of instability if the SLS were used, with prolonged inflation of DCB (multi vessels and left ventricular ejection fraction ≤30%, isolated left main coronary artery).

Patients eligible for a stent‐less strategy underwent lesion preparation with scoring balloon (NSE Alpha, Nipro Europe, Michelen, Belgium) angioplasty (SCBA). If lesion preparation was successful, the second step of angioplasty was performed with DCB (SeQuent Please Neo, B. Braun Melsungen AG, Germany). In case of persistent residual stenosis ≥30%, dissection at high risk of acute occlusion (≥ C [11], and regardless of vessel diameter), or dissection at risk of secondary aneurysmal (≥ B, if it is extensive and occurs in a large‐calibre vessel), bailout stenting with DES was performed (bailout‐DES).

### 2.5 Variables

The endpoint of this analysis was TLR within 36 months of the procedure. In addition, clinical data, procedural data and outcome data were collected.

### 2.6 Statistical Analysis

The stent burden or metal load per patient was determined by calculating the metal index. This allowed classification of patients treated with DCB‐only (metal index of 0), and with bailout‐DES (from an intermediate metal index for patients treated with both bailout‐DES and DCB, to a metal index of 1 for patients treated entirely with DES only). The metal index was calculated by dividing total stent length by total length of lesions treated. Means +/‐ standard deviations or median and interquartile range, counts and percentages were used to describe continuous and categorical variables, respectively. Normal distributions were verified and *T*‐test or Kruskal–Wallis or Mann–Whitney tests were used to compare continuous data of independent samples where appropriate. Chi‐square or Fisher test of homogeneity was used for categorical variables. We used the Kaplan–Meier method to estimate survival probabilities from index procedure until target lesion revascularisation and their pointwise 95% confidence intervals. The Logrank non‐parametric test for comparison of survival distributions was used to compare survival differences between DCB‐only and bailout‐DES groups. An alpha level of 0.05 was used for all statistical tests.

## 3 Results

### 3.1 Study Population

**Figure 1:**
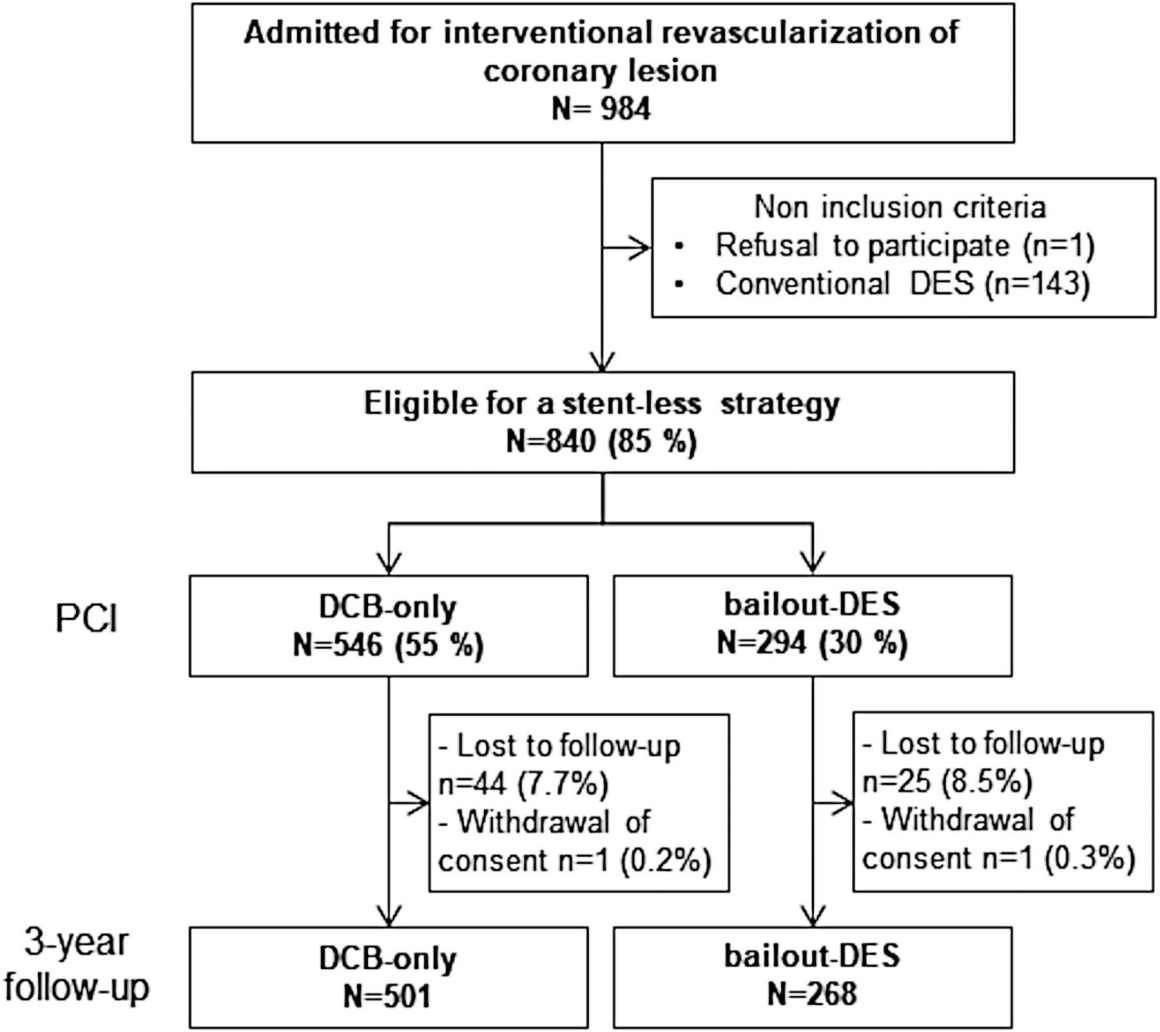
Flow diagram. DCB, Drug Coated Balloon; DES, Drug Eluting Stent; PCI, percutaneous coronary intervention.

From April 2019 to March 2020, 984 consecutive patients underwent interventional revascularization and 840 (85%) were eligible for a stent‐less strategy (Figure 1). DCB‐only was used to treat 546 patients (metal index of 0) and bailout‐DES was required for 294 patients (mean metal index of 0.64). Followed up at 3 years was available for 769 patients revascularized with a stent‐less strategy.

### 3.2 Patient Characteristics

Clinical characteristics and cardiology history were similar in patients in the DCB‐only and bailout‐DES groups, with the exception of family history of coronary artery disease (Table 1). Compared with the bailout‐DES group, chronic coronary syndrome and single vessel disease were more frequent in the DCB‐only group. Conversely, the total lesion length was longer and number of used devices was higher in the bailout‐DES group.

**Table 1:** Characteristics of patients treated with a DCB‐based strategy.

	DCB‐only		Bailout‐DES		*p* value*
	(*n* = 501)		(*n* = 268)		
**Demographics**					
Age, year	68.7	(±11.3)	69.2	(±11.3)	0.768
Men	374	(75%)	201	(75%)	0.915
Mean BMI, kg/m2	27.4	(±4.9)	27.4	(±4.4)	0.831
**Clinical characteristics**					
Family history of coronary artery disease	143	(29%)	46	(17%)	**< 0.001**
Diabetes	118	(24%)	69	(26%)	0.548
High blood pressure	275	(55%)	152	(57%)	0.735
Hypercholesterolemia	239	(48%)	148	(55%)	0.050
Smoking status					
No smoker	242	(49%)	133	(50%)	
Current smoker	88	(18%)	60	(22%)	0.164
Former smoker	166	(33%)	75	(28%)	
**Cardiovascular history**					
Angioplasty	125	(25%)	53	(20%)	0.090
Coronary artery bypass graft	25	(5%)	10	(4%)	0.409
Myocardial infarction	37	(7%)	21	(8%)	0.851
Stroke	9	(2%)	6	(2%)	0.687
Lower limb peripheral arterial disease	27	(5%)	19	(7%)	0.361
**Left ventricular ejection fraction** (*n* = 887)					
≥ 50%	425	(86%)	219	(82%)	
35%–50%	51	(10%)	34	(13%)	0.436
≤ 35%	19	(4%)	13	(5%)	
**Presentation**					
STEMI	55	(11%)	49	(18%)	
NSTEMI	107	(21%)	77	(29%)	**< 0.001**
Chronic coronary syndrome	339	(68%)	142	(53%)	
**Multivessel disease**					
Single vessel	357	(71%)	123	(46%)	**< 0.001**
Multi vessels	144	(29%)	145	(54%)	
**Lesions treated**					
Total length of lesion treated, mm	52.3	(±33.9)	69.7	(±44.6)	**< 0.001**
Total length of stent, mm	0.0	(±0.0)	34.6	(±26.7)	**< 0.001**
Mean diameter, mm	3.1	(±0.4)	3.1	(±0.4)	0.365
Metal index	0.00	(±0.0)	0.64	(±0.3)	**< 0.001**
Patients treated for at least one					
Bifurcation lesion	144	(29%)	115	(43%)	**< 0.001**
Chronic total occlusion	47	(9%)	60	(22%)	**< 0.001**
In‐stent restenosis	76	(15%)	15	(6%)	**< 0.001**
Heavily calcified lesion	26	(5%)	12	(4%)	0.664

* *p* values in bold indicate a significant difference between bailout‐DES and DCB‐only groups.

### 3.3 Concomitant Drug Treatment

More than half of the patients treated with at least one stent were still on a dual antiplatelet therapy at 3 years. The same was observed for patients treated with DCB‐only for acute coronary syndrome. However, the majority of patients treated with DCB‐only for chronic coronary syndrome were on a single antiplatelet therapy (71%).

### 3.4 Target Lesion Revascularization

During the 3‐year follow‐up, the TLR rates were respectively 2.6% and 6% in patients in the DCB‐only and bailout‐DES groups, respectively (OR = 2.38; 95% CI:1.13–5.03; *p* = 0.032). Median metal index was 0 (IQR: 0.36) in patient without TLR and 0.25 (IQR: 0.5) in patients with TLR (*p* = 0.059).

The survival curves for TLR‐free patients in the two groups are shown in the graphical abstract. There was a difference between survival distributions of DCB‐only and bailout‐DES groups (*p* = 0.016). At 12 months, the TLR‐free survival was 99.1% (95% CI: 97.8–99.6) for DCB‐only and 98.3% (95% CI: 95.9–99.3) for bailout‐DES, and at 24 months, the TLR‐free survival was 97.9% (95% CI: 96.3–98.9) for DCB‐only and 95.4% (95% CI: 92.2–97.3) for bailout‐DES.

No association was found between cardiovascular history and TLR, nor between clinical presentations (STEMI, NSTEMI, chronic coronary syndrome) and TLR.

**Figure 2:**
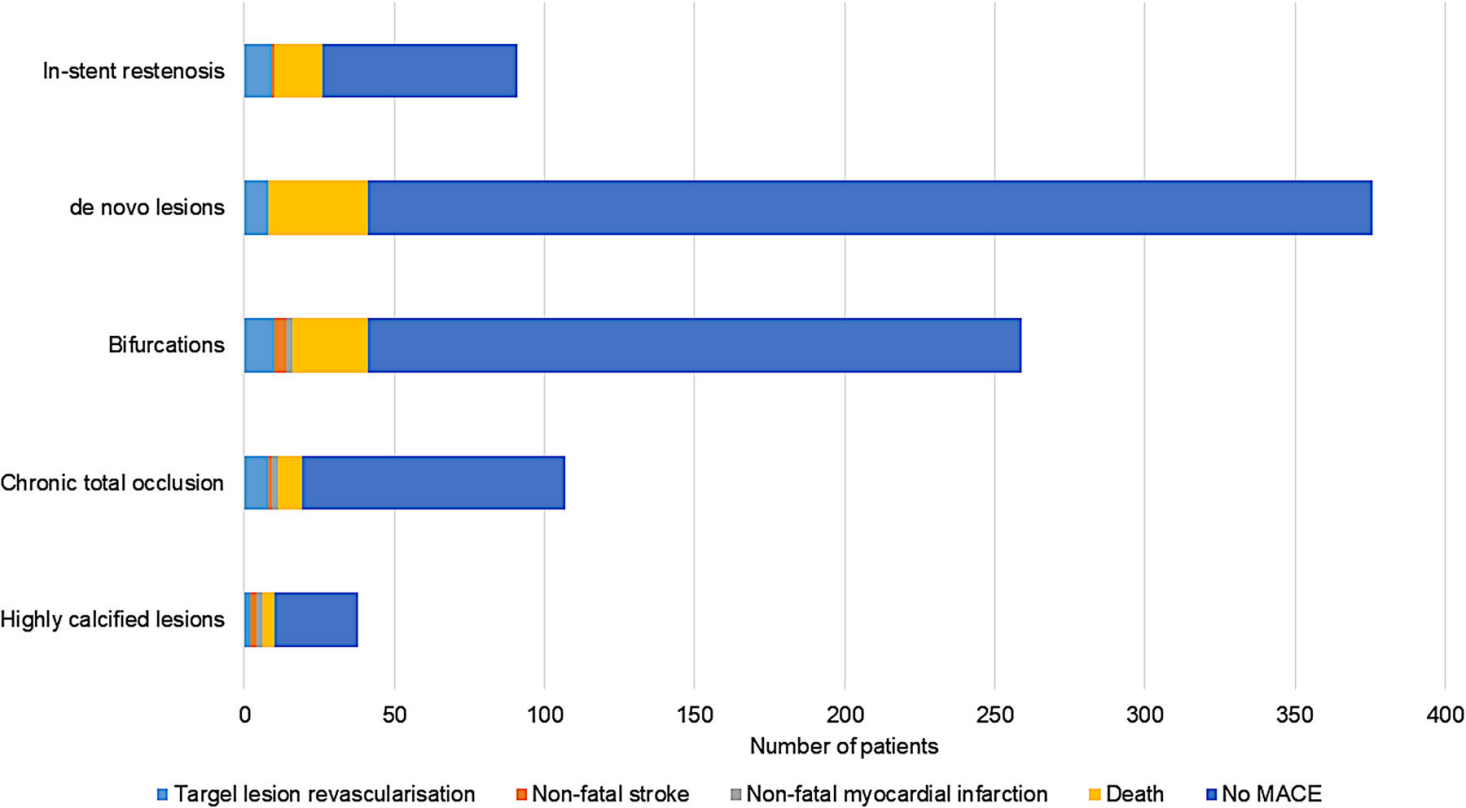
Histogram of patient outcomes by type of lesion treated.

Patient outcomes by type of lesion treated are shown in Figure 2. We found higher rates of TLR in patients treated for at least one chronic total occlusion (OR = 2.47; 95% CI: 1.06–5.72; *p* = 0.049) or at least one in‐stent restenosis (OR = 3.61; 95% CI: 1.59–8.19; *p* = 0.004). We observed no difference in patients treated for a bifurcation lesion or a highly calcified lesion.

## 4 Discussion

### 4.1 Effectiveness of the Stent‐Less Strategy

Revascularisation was effective at 3 years, even more so with DCB‐only, with a low rate of target lesion revascularization (2.6%). This rate is lower than that reported in other groups of patients treated with DCB‐only and with a 3‐year follow‐up (9% in BASKET SMALL 2 [12], 6% in Yang et al. study [13], 9% in Piccoleto‐II [10]). These studies involved patients mainly with de novo lesions in small vessels (˂3 mm). In our study, the diameter of the treated vessel was not associated with the occurrence of target lesion revascularization. This lower rate may be due to lesion preparation using scoring balloons before DCB use in stable patients.

We observed a mortality rate of 10%. This rate is higher than the ones reported in studies of selected populations (> 3% in Yang et al. [13]) or in randomised studies (4% in PICCOLETO‐II [10]). This may be explained by the all‐comers design of the study involving unselected patients. Reduced LVEF is a risk factor for 3‐year mortality, and the lower the LVEF, the greater the risk. The poor prognosis of patients with coronary artery disease and reduced LVEF is consistent with the literature [14].

### 4.2 Lesion Preparation as Part of the Stent‐Less Strategy

The latest Japanese consensus on the use of DCB in coronary artery disease recommends a scoring or cutting balloon for lesion preparation [6]. When successful, this initial preparation results in a residual stenosis of less than 30%, with dissection limited to type B. In the opposite case, particularly in the case of recoil generating a residual stenosis of more than 30%, the risk of restenosis after DCB‐only is nearly 75% [15]. We successfully used the NSE Alpha scoring balloon studied in the Password study [16]. As described by Ilia et al. [17], the balloon should be inflated gradually, at low pressure, allowing the plaque to migrate and limiting the risk of dissection. By controlling inflation progressively and in small steps, scoring should also make it possible to control parietal tension [16]. Other criteria, such as a DCB/vessel ratio of 0.95 and a DCB inflation time of 60 s, are independent factors limiting the occurrence of target lesion revascularization [18].

### 4.3 Indicator of the Stent‐Less Strategy

In our study, we applied a decision algorithm in favour of a stent‐less strategy. The metal index was used to measure the extent to which this strategy has been implemented and to determine the metal load (or stent burden) in a patient. The average metal index of the 769 patients referred to a stent‐less strategy was 0.22, that is, reducing the total stent length that would have been used in a conventional approach by 78%. Similarly, Shin et al. studied the clinical impact of multivessel revascularization using a stent‐less strategy (DCB‐based treatment) versus a conventional DES approach (DES‐only treatment) [9]. The DCB‐based treatment group had a calculated metal index of 0.37, that is the metal load in this group was reduced by 63% compared to the conventional DES‐treated group. This reduction in metal load was associated with a lower rate of MACE and target lesion revascularisation at 2 years (4% vs. 11%, and 3% vs. 6%, respectively). The higher rates in the DES‐only treatment may be explained by very late stent‐related events with an annual incidence of MACE of 2%, such as stent thrombosis or neo‐atherosclerosis, as observed by Madhavan et al [19]. This risk increases with the length of stents [20].

### 4.4 Limitations of the Study

This study evaluates the practices of five interventional cardiologists with different levels of experience, but it is a single‐centre study and its external validity is therefore limited. The low restenosis rate required the use of less powerful non‐parametric tests. About 8% of patients were lost to follow‐up, which could bias the rate of MACE. It is difficult to evaluate the respective contribution of stent or dual antiplatelet therapy, as this study, designed in 2018, has several limitations: the antiplatelet therapy regimens applied were very heterogeneous. The single antiplatelet therapy (P2Y12 inhibitor) was not yet routinely prescribed for DCB‐only patients, and we observed that more than half of the patients treated with at least one stent were still on a dual antiplatelet therapy at 3 years. The study focused on ischemic risk without investigating the bleeding risk caused by major bleeds, and was unable to characterize the ischemic/haemorrhagic balance of NACE (Net Clinical Adverse Events), as in the TICO [21] and TWILIGHT [22] studies. It is therefore difficult to establish the effect of prolonged antiplatelet therapy, as mandated by the current ESC guidelines, on the observed mortality rate.

## 5 Conclusions

The SLS based on a combination of scoring balloon and DCB was effective at 3 years. The metal index could be used to measure the extent to which the SLS has been implemented and to determine the metal load (or stent burden) in a patient.

## Ethics Statement

This study complies with the Declaration of Helsinki. It was approved by the ethics committee (18.12.26.53103, CPP Sud‐Est VI).

## Consent

Informed consent was obtained from all individual participants included in the study before the revascularization procedure, or afterwards in case of emergency. A letter explaining the study and the patients’ rights regarding the use of their data was given to the patients.

## Conflicts of Interest

Ludovic Meunier reports grants and personal honoraria payment from B Braun. Virginie Laurençon and Caroline Allix‐Béguec reports grants from B Braun. Matthias Waliszewski is an employee of B Braun. The other authors declare no conflicts of interest.

## Data Availability

The data underlying this article are available in Zenodo (https://doi.org/10.5281/zenodo.10036350).
